# System for the remote control and imaging of MW fields for spin manipulation in NV centers in diamond

**DOI:** 10.1038/s41598-020-61669-w

**Published:** 2020-03-16

**Authors:** Giacomo Mariani, Shuhei Nomoto, Satoshi Kashiwaya, Shintaro Nomura

**Affiliations:** 1 Division of Physics, University of Tsukuba, Tennodai, Tsukuba, Ibaraki 305-8571 Japan; 2 Department of Applied Physics, Nagoya University, Chikusa-Ku, Nagoya 464-8571 Japan

**Keywords:** Imaging techniques, Nanophotonics and plasmonics, Sensors, Imaging and sensing

## Abstract

Nitrogen-vacancy (NV) centers in diamond have been used as platforms for quantum information, magnetometry and imaging of microwave (MW) fields. The spatial distribution of the MW fields used to drive the electron spin of NV centers plays a key role for these applications. Here, we report a system for the control and characterization of MW magnetic fields used for the NV spin manipulation. The control of the MW field in the vicinity of a diamond surface is mediated by an exchangeable lumped resonator, coupled inductively to a MW planar ring antenna. The characterization of the MW fields in the near-field is performed by an FFT imaging of Rabi oscillations, by using an ensemble of NV centers. We have found that the Rabi frequency over a lumped resonator is enhanced 22 times compared to the Rabi frequency without the presence of the lumped resonator. Our system may find applications in quantum information and magnetometry where a precise and controlled spin manipulation is required, showing NV centers as good candidates for imaging MW fields and characterization of MW devices.

## Introduction

The coherent manipulation of the electron and nuclear spin in nitrogen-vacancy (NV) centers in diamond has become fundamental for both quantum information processing and sensing applications^1,2^. Single and double quantum bits (qubits) are realized in NV centers by driving single spins with resonant microwave (MW) or radio-frequency (RF) fields and specific pulse sequences^3–6^. Ensembles of NV centers have proved to be excellent magnetometers,^7–10^ offering high spatial resolution with a signal-noise ratio proportional to $\sqrt{{N}_{NV}}$, where *N*_*NV*_ is the number of the driven spins. For these applications, a precise spatial control of the MW field distribution is required to coherently drive single or ensembles of spins. In imaging and sensing applications, the commonly employed MW antennas have a large bandwidth and can generate uniform MW fields in a wide area^11,12^ or in a 3D volume^13,14^. For quantum information, the area of interest is limited to few or single centers, for which high and more localized MW magnetic fields are preferred to drive the spin in an efficient way. For instance, miniaturized MW loops^15^, thin wires, and coplanar waveguides^16^ fabricated directly on the diamond surface offer higher magnetic field amplitude in the near-field but they can be easily subjected to disconnections or induce undesired sample heating.

Here, we demonstrate a simple system for the control and characterization of the MW magnetic field to be employed for spin manipulation in NV centers in diamond at room temperature. The MW field is generated by excitation of a gold lumped resonator coated on a silicon substrate; the resonator and its MW field distribution can be designed according to the specific application. For the excitation of the lumped resonator we use a large MW planar ring antenna, which provides a uniform MW field over the sample at a distance of about 0.5 mm. The resonator, in close proximity of the diamond surface, is coupled inductively to the MW antenna and its re-emitted MW field is then sensed by NV centers. Compared to previous systems operating at room temperature, the lumped resonator is not fed directly by electrical current and can be easily substituted without the need of electrical connections. The MW field distribution over the resonator can be quickly measured by using an ensemble of near-surface NV centers in the same diamond substrate, performing the imaging of the MW magnetic field in the near-field. Ensembles of NV centers, which have been previously used for imaging MW magnetic fields^17–19^, offer a fast and precise way of MW imaging. The imaging method consists in driving the electron spin with the MW field and measuring the frequency of Rabi oscillations, which is directly associated with the MW field intensity of the external field. This method gives a quantitatively accurate measurement of the MW field intensity.

## Results

### Optically detected magnetic resonance spectrum of diamond NV centers

**Figure 1:**
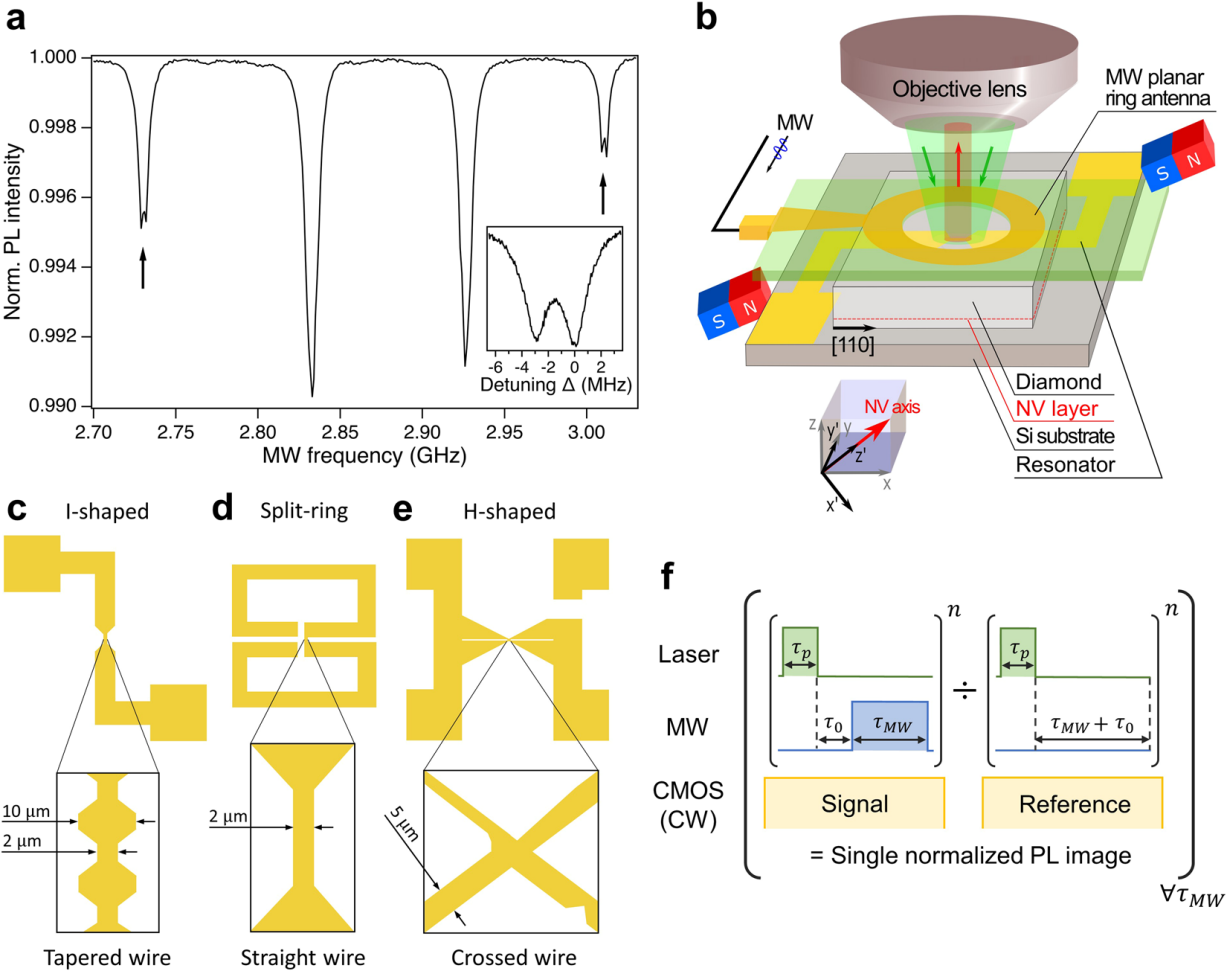
(**a**) Pulsed-ODMR spectrum of NV centers acquired at a MW power of 14.3 dBm and a MW *π*-pulse duration of 1 *μ*s, with a static magnetic field *B*_0_ = 4.6 mT aligned along the [111] direction. The two black arrows indicate the transitions $\left|0\right\rangle$ → $\left|\pm 1\right\rangle$ correspondent to the [111] direction used in this experiment to perform the MW magnetic field imaging. The two inner resonance peaks correspond to the other three possible orientations of the symmetry axis of the NV centers. The inset shows a zoom of the peak at 3.010 GHz which shows a splitting associated with the hyperfine interaction of ^15^N nuclear spin at a MW power of 9.3 dBm and a MW pulse duration of 1.5 *μ*s. (**b**) Experimental setup used for the excitation of the resonator and the imaging of the MW magnetic field distribution. The green laser at *λ* = 520 nm is focused through an objective lens 100 ×  on the NV layer. (**c**–**e**) Sketches of the lumped resonators in the *xy*-plane that we measured with our system. The zoomed insets show the area of the patterns over which we performed the MW imaging. (**f**) Pulse sequence used to measure the Rabi oscillations of the electron spin.

An NV center in diamond is a defect in the diamond lattice constituted by a vacancy and an adjacent nitrogen atom which substitutes a carbon atom. The ground state of NV centers is a spin-triplet whose singlet state *m*_*s*_ = 0 and doublet state *m*_*s*_ =  ± 1, named here $\left|0\right\rangle$ and $\left|\pm 1\right\rangle$, have a transition (zero-field splitting) at a frequency of 2.87 GHz, which makes them ideal for imaging MW fields. The degeneracy of the states $\left|\pm 1\right\rangle$ is lifted by a static external magnetic field *B*_0_ which produces a Zeeman energy splitting of 2*γB*_0_, where *γ* is the gyromagnetic ratio of the electron spin. The spin-state transitions are characterized by optically detected magnetic resonance (ODMR)^20^. The spin state is optically initialized by an off-resonance green laser pumping it in the state $\left|0\right\rangle$. After the manipulation with a MW field resonant with the transitions $\left|0\right\rangle$ → $\left|\pm 1\right\rangle$, the spin state is measured through the photoluminescence emitted in a spectral range of *λ* = 630-800 nm. The static magnetic field *B*_0_, employed to remove the degeneracy of the states $\left|\pm 1\right\rangle$, produces eight magnetic resonances correspondent to the four possible orientations of the symmetry axis of the NV centers, [111], [− 11− 1], [1 − 1−1], and [− 1–11], and two spin transitions $\left|0\right\rangle$ → $\left|\pm 1\right\rangle$. In principle, measuring the MW field projection along the four possible orientations, it would be possible to fully reconstruct the external MW magnetic field vector^8,21^. In our case, we aligned *B*_0_ ≃ 4.6 mT along the [111] direction. The resonance transition of the NVs oriented along the [111] direction was used to map the MW field distribution on the resonator. In this case, the projections of *B*_0_ along the other possible NV orientations are the same, leading to four magnetic resonances to appear in the ODMR spectrum, two for the spin transitions of the NVs oriented along the [111] direction and two for the other directions, as shown in Fig. 1(a). The hyperfine interaction between the electron spin and the ^15^N nuclear spin (*I* = 1∕2) causes an additional energy splitting at *A*_‖_ = 3 MHz^22^, as shown in the inset of Fig. 1(a).

### Microwave imaging apparatus and lumped resonators

Figure 1(b) shows a pictorial representation of our experimental setup based on a wide-field optical microscope. The pulsed laser diode used for the excitation of NV centers operates at a wavelength of *λ* = 520 nm. The photoluminescence arising from the diamond chip is imaged by a microscope equipped with a cooled scientific CMOS camera. The static magnetic field is used to lift the degeneracy of the states $\left|\pm 1\right\rangle$. The core of our measurements is a (100) diamond chip with an ensemble of NV centers located at a depth of  ~ 10 nm from the surface, employed as a platform for the MW field imaging. The diamond substrate is sandwiched between a MW planar ring antenna and a gold lumped resonator. Figure 1(c–e) show a sketch of the three resonators we employed to demonstrate our system. The MW planar ring antenna is a single-loop coil surrounding a circular hole with a radius of 0.5 mm^23^. It provides a spatially uniform magnetic field in an area of 0.785 mm^2^. The coupling with the lumped resonators is mediated by the magnetic field of the MW antenna which penetrates their central wire.

The resonators have a resonance frequency in a range of a few GHz and their common characteristic is a central thin wire which we used to drive the electronic spin in NV centers. Other structures can be realized by appropriate design of the lumped inductors, capacitors, and lossy elements. However, here we don’t analyze the design of the resonators since it goes beyond the scope of this work. We fabricated the central wires with different shapes: tapered (Fig. 1(c)), straight (Fig. 1(d)) and crossed (Fig. 1(e)). In the first two cases, the wires are used to control the MW field amplitude by a highly localized density current. The change in the width of the tapered wire is used to spatially control the amplitude of the MW magnetic field. The crossed-wire sample has a more complicated pattern which we used to show the spatial resolution of the image and the possibility of our system to sense circularly polarized MW fields. Note that with respect to other reported lumped MW antennas e.g.^11^, in this case the main MW source is fixed and the resonating circuit can be replaced with other patterns. It should be noted that an advantage of this particular configuration is that we can perform measurements even far from the central wires of the lumped resonators since the MW field does not drop to zero. In fact, the conventional direct feed of a coplanar waveguide^16^, for example, would limit the measurement area in the near-field of a MW current. In our system, without the need to remove the sample, we effectively perform measurements even on a wide sample area far from the wires of the resonators where the MW field is determined only by the homogeneous MW field generated by the MW planar ring antenna.

For the characterization of the lumped MW resonators, we performed the imaging of the MW magnetic field distribution over their central wires, by using a dense ensemble of NV centers in diamond. According to the selection rules, the transitions between the states $\left|0\right\rangle$ and $\left|\pm 1\right\rangle$ are sensitive to circular polarization^24^ and in a rotating wave approximation (RWA) are allowed only for circularly polarized MW fields. The MW field to be imaged, in resonance with the spin transitions $\left|0\right\rangle$ → $\left|\pm 1\right\rangle$, drives Rabi oscillations between the two levels with Rabi frequencies Ω_0_∕2*π* = *γB*_±_, where *γ* = 28 GHz/T is the electron gyromagnetic ratio and *B*_+(−)_ is the amplitude of the left (right) handed circularly polarized amplitude of the MW field. The Rabi frequency is directly proportional to the amplitude of the magnetic field. Hence, the imaging of the MW field at a certain position is performed by measuring the Rabi oscillations and calculating the related frequency by Fast Fourier Transform (FFT). The components of the MW field in the plane perpendicular to the NV axis drive the Rabi oscillations of the electron spin and thus are sensed by the system.

The external magnetic field *B*_0_ should be strong enough to separate the resonance that results from NVs oriented along the [111] direction from the resonance peaks of NVs oriented along the other directions. In fact, for large MW powers, the MW field parallel to the [111] direction could drive Rabi oscillations of close resonance transitions, which introduce multiple components at higher frequency in the measurement. We used a maximum MW power of 37.3 dBm, since at this power we started to observe these components in the FFT spectra.

The Rabi oscillations are measured by using the pulse sequence depicted in Fig. 1(f). The sequence to measure the Rabi oscillations at a specific MW pulse duration *τ*_*MW*_ starts with a green laser pulse with a duration of *τ*_*p*_ = 1 *μ*s which prepares the spin in the $\left|0\right\rangle$ state. After a waiting time of *τ*_0_ = 1 *μ*s, necessary to complete the spin polarization, the electron spin is driven by a MW pulse resonant with $\left|0\right\rangle$ → $\left|\pm 1\right\rangle$; the spin state is measured by applying a second laser pulse of the same duration, which reinitializes the spin state in $\left|0\right\rangle$. For a fixed MW pulse duration *τ*_*MW*_, the previous sequence is repeated *n* = *p* ⋅ *q* cycles, where *p* are dozens of thousands of cycles, for a total time equal to the integration time of the CMOS camera which works in a continuous (CW) mode and *q* is the number of repetitions of a single measurement which is usually repeated a few hundred times and averaged. The signal is normalized by a reference acquired by the same sequence with the MW pulse off. The output is a single normalized PL image of the CMOS camera with a size of 34.9 × 33.9  *μ*m^2^ (528  ×  512 pixels). To temporally reconstruct the Rabi oscillations, the previous procedure is performed again for increasing MW pulse durations. At the end of the measurement we obtain several images of the CMOS camera corresponding to different MW pulse durations. The Rabi oscillations at a specific position are calculated by binning a small area of *N* × *N* pixels in the images acquired by the CMOS camera, with e.g. *N* = 2, 4, 8. On the one hand, the binning improves the signal to noise ratio, mitigating mechanical vibrations and thermal oscillations of the optical elements of the experimental setup during a long measurement. On the other hand, it limits the spatial resolution of the measurement but it is still sufficient to reconstruct the MW field distribution over the lumped resonators. The frequency of the Rabi oscillations of *N* × *N* pixels is calculated by performing the FFT and identifying the maximum intensity peak in the spectrum. The diffraction limit of our imaging evaluated at a wavelength *λ* ≃  700 nm is limited by the numerical aperture of the objective lens NA  = 0.73 at *λ*/(2 ⋅ NA)  ≃  480 nm, which is larger than our minimum imaging pixel size of 66 nm for *N* = 1. The possibility to discriminate between two magnetic point sources is then limited by the standoff distance between the NV layer and the sample surface in range of  ≃  1.25–3 *μ*m with our NV layer implanted at a depth of about 10 nm.

Note that due to the splitting caused by the ^15^N nuclear spin, as shown in the inset of Fig. 1(a), we have to select one of the two resonance peaks. Considering one of the two peaks, a MW field off-resonance would drive Rabi oscillations with the general Rabi frequency ${\Omega }^{{\prime} }=\sqrt{{\Omega }_{0}^{2}+{\Delta }^{2}}$, where Ω_0_ is the Rabi frequency on-resonance and Δ is the detuning off-resonance. This means that a MW field resonant with one of the two transitions would make a double frequency to appear in the FFT spectrum of the Rabi oscillations for frequencies close to *A*_∣∣_ = 3 MHz. A simple and effective solution is to choose the MW frequency of the driving field at the center of the nuclear spin splitting with Δ ≃  1.5 MHz. In this case, we take the same contribution in frequency detuning from the two resonances, avoiding beats in the Rabi oscillations. In the case of ^14^N isotopes with three resonance peaks, this is not possible and a beat would appear in the Rabi oscillations (see e.g. Wang *et al*.^19^). In our system, the minimum Rabi frequency sensed in the measurements is limited by Δ and only for ${\Omega }_{0}^{2}\gg {\Delta }^{2}$ we assume ${\Omega }^{{\prime} }\simeq {\Omega }_{0}=2\pi \gamma$B_±_, for which the MW field depends linearly on the general Rabi frequency.

### Imaging of MW field distribution

In this section we show the experimental results of the MW imaging performed over the patterns shown in Fig. 1(c–e) and the comparison with their simulated MW field distributions. The MW magnetic field as measured by NV centers is calculated by first transforming the frame of reference of the laboratory $\left\{x,y,z\right\}$ in a new set of coordinates $\left\{{x}^{{\prime} },{y}^{{\prime} },{z}^{{\prime} }\right\}$, where the ${z}^{{\prime} }$-axis is aligned along the [111] NV-axis (Fig. 1(b)). The circularly polarized amplitude of the MW field sensed by NV centers is calculated as

1 $${B}_{\pm }=\left|B\widehat{{x}^{{\prime} }}\mp iB\widehat{{y}^{{\prime} }}\right|\ ,$$

where + (−) is the left (right) handed component of the circularly polarized field and $\widehat{{x}^{{\prime} }}$ and $\widehat{{y}^{{\prime} }}$ are the unit vectors in the new NV frame. The simulations are performed by finite-difference time-domain (FDTD) analysis. In the simulation, the source of the MW field is a double dipole antenna located at a distance of 30 mm from the resonators and the dipoles are phase-shifted by 180°. This system ensures a uniform and linearly polarized magnetic field along the direction perpendicular to the surface of the resonators (*z*-axis, in the lab frame). The excitation source in the simulation is chosen as a simple approximation of the real MW antenna, which generates a homogeneous MW field along the *z*-axis within its central aperture. Since the components of the MW field along the *x*-axis and *y*-axis are negligible, and the modelled dipoles are much larger than the size of the sample, we consider them a good approximation of the MW planar ring antenna.

**Figure 2:**
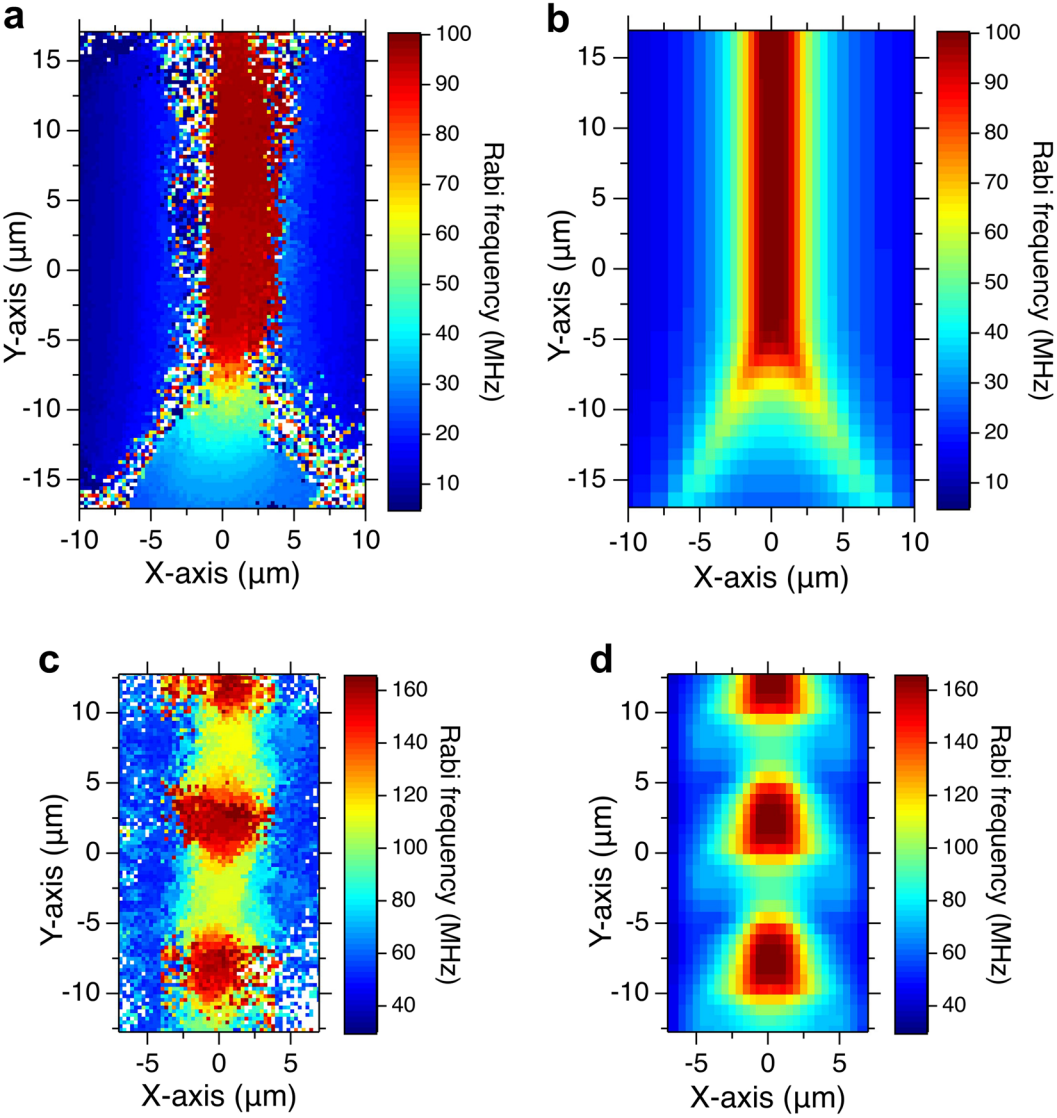
Comparison between the MW imaging and FDTD simulation for the resonators with a straight wire and a tapered wire. The single points of the measurements are calculated by averaging an area of 4 × 4 pixels. (**a**) MW imaging of the straight-wire resonator and its simulation in (**b**), performed at a frequency of 2.945 GHz ($\left|0\right\rangle$ → $\left|+1\right\rangle$). (**c**) MW imaging of the tapered-wire resonator and its simulation in (**d**), performed at a frequency of 2.7309 GHz ($\left|0\right\rangle$ → $\left|-1\right\rangle$). The MW imaging of both resonators is performed at a MW power of 35.3 dBm. The white points in (**a**) and (**c**) are noisy points which are discarded from the image through a threshold applied to the FFT amplitude.

Figure 2 shows a comparison between the measurement and simulated results of the MW imaging of the tapered and straight wires performed at a MW power of 35.3 dBm. The gold film has a thickness of 100 nm, much thinner than the skin depth of 1.38 *μ*m, calculated at a frequency of 3.000 GHz, so the current density is homogeneously distributed across the wire thickness. As shown in Fig. 2(a), the MW field over the straight wire is nearly uniform along its length and confined within the wire width of 2 *μ*m, reaching a maximum Rabi frequency of  ~ 100 MHz. In the case of the tapered wire shown in Fig. 2(c), the MW field intensity is controlled as a function of the wire width (2–10  *μ*m) and the Rabi frequencies reach a maximum of  ~ 165 MHz in the narrowest part of the wire. We compared the maximum Rabi frequencies recorded above the straight and tapered wires with the bulk Rabi frequency acquired at the same MW power of 35.3 dBm, in the presence of only the MW planar ring antenna without resonators. We achieved a 20-fold enhancement of the Rabi oscillations for the straight wire and a 22-fold enhancement for the tapered wire. The FDTD simulations plotted in Fig. 2(b,d) show a good agreement with the measured MW field distributions. Comparing the experimental data with the simulation, the distance between the NV layer and the surface of the wires has been estimated as  ~ 1.50 *μ*m for the tapered wire and  ~ 1.25 *μ*m for the straight wire. The short distance between the NV layer and the gold wires is crucial to obtain high Rabi frequencies and thus a clean sample surface and the correct positioning of the sample play a key role. The MW field enhancement in the vicinity of the wires can be viewed in terms of dipole emission from the wire, for which the magnetic field is written as

2 $${\bf{B}}({\bf{r}})=\frac{{\mu }_{0}}{4\pi }\left[-\frac{{\bf{r}}\times \dot{{\bf{p}}}}{{r}^{3}}-\frac{{\bf{r}}\times \ddot{{\bf{p}}}}{{r}^{2}}\right]\ ,$$

with

3 $$\dot{{\bf{p}}}({t}^{{\prime} })=\int {{\bf{i}}}_{{\bf{w}}}({{\bf{r}}}^{{\prime} },{t}^{{\prime} }){d}^{3}{{\bf{r}}}^{{\prime} }\ ,$$

where **i**_**w**_ is the current density flowing in the wire. The magnetic field increases rapidly as  ∝ *r*^−2^ in the near-field, in close proximity of the wire surface.

**Figure 3:**
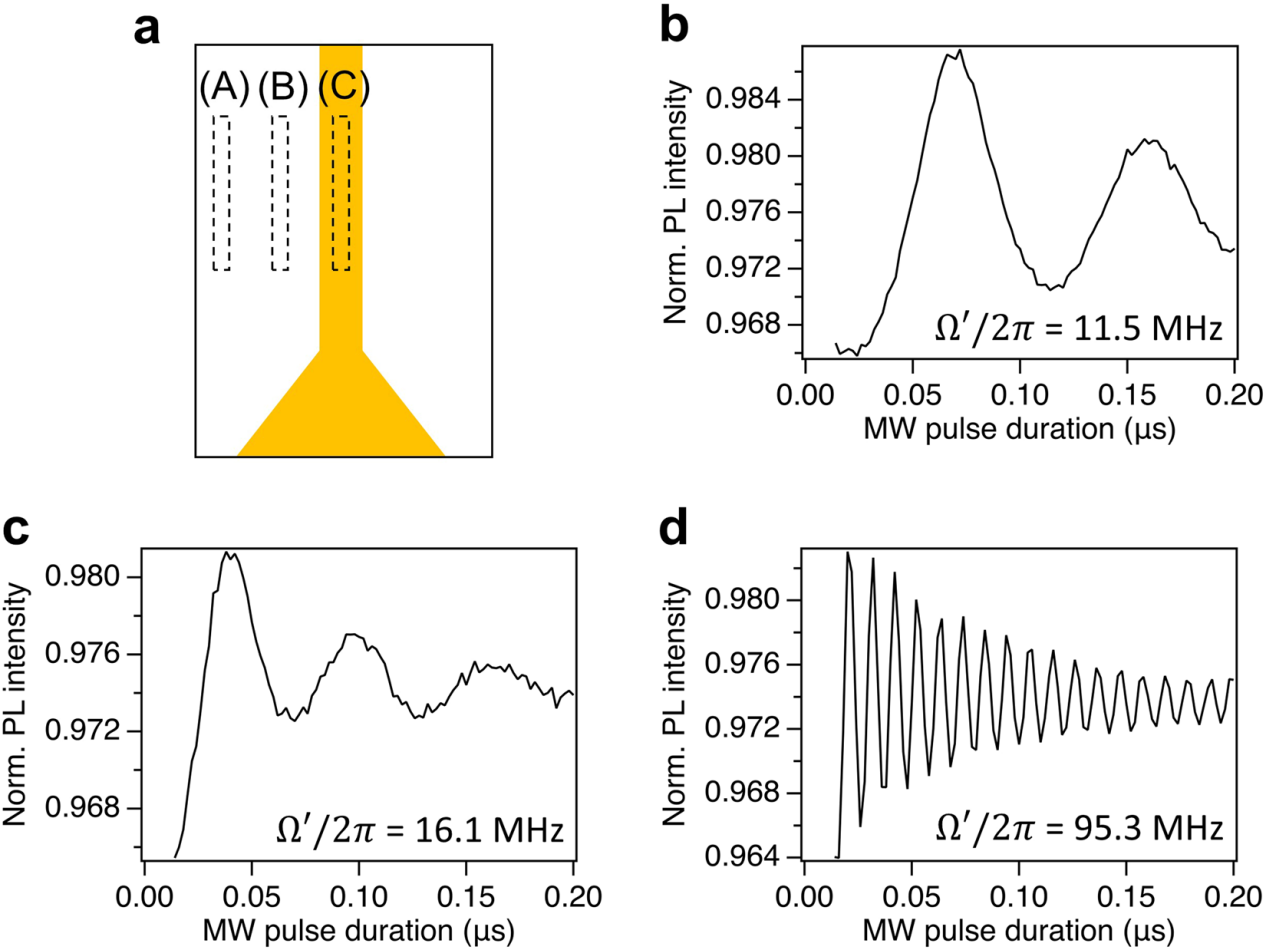
(**a**) Sketch of the straight wire. The dashed rectangles A-C indicate the area of the measured Rabi oscillations. Rabi oscillations in the area (**b**) A, (**c**) B, and (**d**) C around the resonator shown in Fig. 1(d).

 Figure 3 displays an example of a measurement of the Rabi oscillations for the imaging of the MW field distribution shown in Fig. 2(a). The Rabi oscillations are acquired for a MW pulse in a range of 0.014–0.2  *μ*s at a step of 2 ns. The measured Rabi oscillation frequency is the fastest in the center region C, and is slower in the region B, and the slowest in the region A, where the distance between the measured area and the wire is the largest.

**Figure 4:**
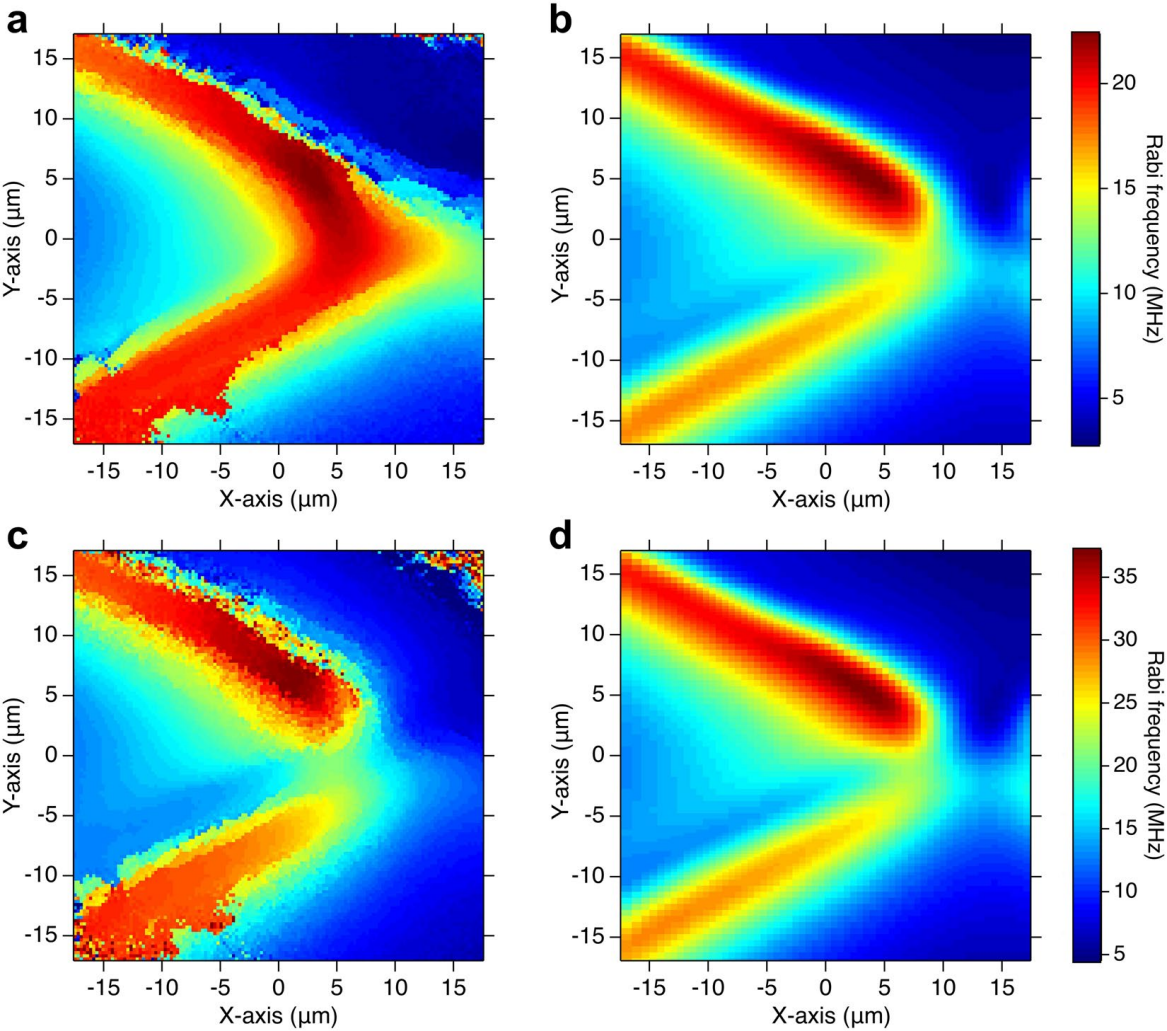
Comparison between the measured Rabi frequency distributions of the crossed-wire resonator at a MW power of 29.3 dBm and the corresponding FDTD simulations. The single points of the measurements are calculated by binning 4 × 4 pixels. (**a**) MW imaging at a frequency of 3.010 GHz ($\left|0\right\rangle$ → $\left|+1\right\rangle$) and its simulated result in (**b**). (**c**) MW imaging at a frequency of 2.730 GHz ($\left|0\right\rangle$ → $\left|-1\right\rangle$) and its simulated result in (**d**). The MW field distribution was calculated on the plane at a distance of 3.0 *μ*m from the surface of the resonator.

The MW imaging performed with NV centers has the possibility to sense circularly polarized fields, providing additional information on the system to be characterized. We show a polarization-sensitive imaging of a crossed-wire resonator, which shows several defects and asymmetries produced during its fabrication process that may confer it a circularly polarized distribution of the MW field. We measured the MW magnetic field distribution at a frequency of 2.730 GHz for $\left|0\right\rangle$ → $\left|-1\right\rangle$ and at a frequency of 3.010 GHz for $\left|0\right\rangle$ → $\left|+1\right\rangle$, for different powers fed to our MW planar ring antenna. Figure 4 shows the imaging of the Rabi frequency distribution measured at a MW power of 29.3 dBm for the $\left|0\right\rangle$ → $\left|\pm 1\right\rangle$ transitions and the corresponding FDTD simulations. We compared the simulated 2D images with the measurement at a few steps from the surface of the resonator in a range of 0.5-3.5 *μ*m, for the $\left|0\right\rangle$ → $\left|-1\right\rangle$ transition. We compared quantitatively the Rabi frequencies in a few points of two images and we matched the profile of the Rabi frequency distribution across the wires. Among the simulated results, we chose the most representative MW field distribution at a distance of 3.0 *μ*m from the surface of the lumped resonator. The measurements in Fig. 4(a,c) show a maximum Rabi frequency of 22.54 MHz (37.18 MHz) above the crossed wires for the transition $\left|0\right\rangle \to \left|+(-)1\right\rangle$. The different MW field distributions for the two spin transitions suggest the possibility that the MW field emitted by the crossed-wire resonator is partially circularly polarized. These results are similar to a crossed-shaped antenna proposed in ref. ^25^, in which circularly polarized fields are used to selectively manipulate the NV spin states. In our case, the different Rabi frequency distributions are due to imperfections in the geometry of the resonator. Observing the images of the sample, we found that the crossed-wire pattern was not symmetric with respect to its central axis due to imperfections in the structure, such as holes and peeled-off areas. To obtain a reconstruction of the measured MW field distribution for the sample with defects, we inserted reproducible planar defects into the FDTD simulation such as cuts or missing parts. However, we did not include three-dimensional defects in the simulation originating from the peel off of the gold film difficult to be reproduced. The simulated distributions in Fig. 4(b,d) reproduce the main features of the measurements and they show, although small, the rise of a circular polarization. We attributed the remaining disagreement between the measured images and the simulated results to our simplified modelling of the sample with defects and to the limited mesh size (0.5 *μ*m) of the FDTD simulation. This result proves the advantage of NV centers in the characterization of MW circuits, with respect to simplified theoretical analysis by FDTD simulation which cannot fully provide information about a real situation. Furthermore, sample characterization by using NV centers in our system can be generally performed in a few minutes, as compared to a single FDTD simulation that requires from several hours up to a few days to be completed. To quantitatively compare the circularly polarized fields, we performed a calibration of the Rabi oscillations with respect to the gain of the MW antenna at the frequencies and MW powers of the measurements. We used as a reference, the bulk Rabi oscillations measured only in the presence of the MW planar ring antenna and we found a ratio of 1.84 between the Rabi frequency measured for the $\left|0\right\rangle$ → $\left|-1\right\rangle$ and the $\left|0\right\rangle$ → $\left|+1\right\rangle$ transitions. Note here that the apparent difference in the Rabi frequency between the transitions $\left|0\right\rangle \to \left|+1\right\rangle$ and $\left|0\right\rangle \to \left|-1\right\rangle$ may be partly explained by a non-flat response of the resonator. Further investigations are necessary to evaluate the response of the resonator to circularly polarized MW field.

**Figure 5:**
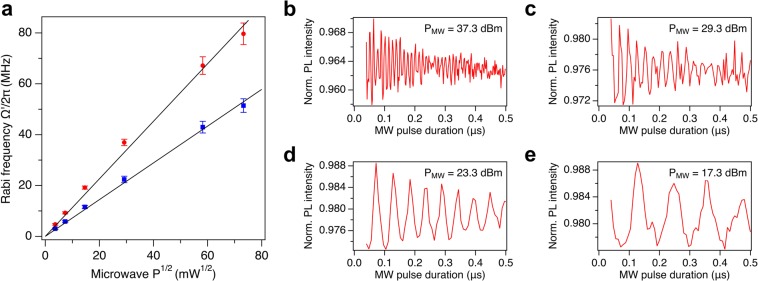
(**a**) Dependence of the general Rabi frequency ${\Omega }^{{\prime} }$ on the square root of the MW power fed to the MW planar ring antenna for the transition $\left|0\right\rangle$ → $\left|-1\right\rangle$ (red circles) and $\left|0\right\rangle$ → $\left|+1\right\rangle$ (blue squares). Considering the images of the Rabi frequency distribution with a size of 132 × 128 pixels, the values are calculated by averaging the first 8 pixels near the pixel with the maximum Rabi frequency, for an area of the size of  ~ 0.63 *μ*m^2^. The error bars are calculated from the standard deviation of the previous average and added to the half width half maximum (HWHM) value of the peak in the FFT spectrum. The black line is a linear fit which shows the linear dependence of ${\Omega }^{{\prime} }$ on $\sqrt{P}$. The right panel shows the Rabi oscillations extracted from (**a**), measured in the point with maximum frequency above the crossed wires of the resonator for the transition $\left|0\right\rangle$ → $\left|-1\right\rangle$, at the following MW powers: (**b**) 37.3 dBm, (**c**) 29.3 dBm, (**d**) 23.3 dBm and (**e**) 17.3 dBm.

We measured the maximum Rabi frequency above the crossed-wire area for different MW powers (11.3–37.3 dBm). Figure 5(a) depicts the linear dependence of the Rabi frequency on the square root of the MW power $\sqrt{{P}_{MW}}$ fed to our MW planar ring antenna. As explained previously, even if we measure a general Rabi frequency, the linear relation is preserved in the limit of fast enough Rabi oscillations. The different slope for the transitions $\left|0\right\rangle$ → $\left|\pm 1\right\rangle$ is due to a different gain of the MW antenna at the frequency of the transitions. In Fig. 5(b–e), we compare the Rabi oscillations measured at different MW powers for the transition $\left|0\right\rangle$ → $\left|-1\right\rangle$. The increasing decay for higher powers is mainly due to the random fluctuations in the power of the MW source^26^ and this problem could be overcome by applying a decoupling sequence such as the concatenated continuous driving scheme^27^.

**Figure 6:**
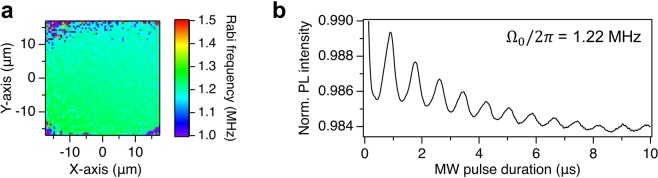
(**a**) Imaging of the bulk Rabi frequency distribution far from the crossed wires, on-resonance with one of the peaks originated from the hyperfine interaction with ^15^N nuclear spin. The position of the FFT peak is determined after a zero-filling of 10 times the measured Rabi oscillations to resolve a minimum shift of 10 kHz. (**b**) Bulk Rabi oscillations with frequency *Ω*_0_/2*π* = 1.22 MHz calculated by averaging the area in (**a**).

Even in the case of the crossed-wire resonator, the MW field is enhanced on the micrometer-scale by means of a high density current localized in the thin wires, as compared to the bulk MW field generated only by the MW planar ring antenna on the millimeter-scale. To obtain a quantitative value of the MW field enhancement, we compared the maximum Rabi frequency measured at a MW power of 29.3 dBm for the transition $\left|0\right\rangle$ → $\left|+1\right\rangle$ with the bulk Rabi frequency in the presence of only the MW planar ring antenna. In this case, the frequency of the bulk Rabi oscillations is comparable with the nuclear spin splitting frequency. We then performed the measurement for a frequency detuning of Δ = 0 (see the inset of Fig. 1(a)), resonant with one of the spin split transitions due to the hyperfine interaction to clearly determine the Rabi frequency. As shown in Fig. 6, the distribution of the bulk Rabi frequency is homogeneous with a frequency of Ω_0_∕2*π* ≃  1.22  ±  0.04 MHz in the area of the imaging. Comparing this result with the maximum frequency measured in Fig. 4(a), we estimate a MW magnetic field enhancement of about 19 times larger and localized in a micrometer-scale area for the resonator.

## Discussion

We have demonstrated a system for the characterization and control of the MW magnetic field distribution emitted by lumped resonators operating in the GHz regime by using an ensemble of NV centers in diamond. The lumped resonators used to drive the NV spins were coupled inductively to a large MW planar ring antenna at a distance of 0.5 mm, without a direct feed of electrical current. The lumped resonators fabricated on a silicon substrate could be easily substituted according to the specific application. We measured the distribution of the MW magnetic field of three different lumped resonators by driving the Rabi oscillations of the electron spin in NV centers, demonstrating that NV centers are a powerful tool for the direct imaging of MW fields. The measurement of the Rabi frequency, directly proportional to the MW magnetic field amplitude, allows us to quantitatively evaluate the MW field distribution over the resonators. For fast spin driving applications, the MW field could be enhanced in the near-field of the thin wires at the center of the resonators. In the case of the shown resonators with straight, tapered, and crossed wires, the maximum Rabi frequency over the thin wires is enhanced 20, 22, and 19 times respectively, compared to the bulk Rabi frequency without resonators. In the resonators, the enhanced MW field is localized in an area on the scale of the minimum width of the thin wires (~2 *μ*m) compared to the MW field generated by the MW planar ring antenna, distributed in an area of the size of 0.785 mm^2^. This enables us to locally drive the electron spins of NV centers, avoiding thermal excitation of unwanted electrons. Our system is particularly advantageous in manipulating NV spins in diamond at a cryogenic temperature using a MW planar ring antenna outside of a cryostat. The complexities due to the wiring and the heat inflow through a MW cable can be eliminated, which is beneficial when one uses a standard ^4^He cryostat for micro-photoluminescence measurements with small space for inserting a MW cable and a MW connector. We believe that our system, in which lumped resonators are coupled remotely to a main antenna, would be a powerful and versatile tool for the coherent manipulation of NV spins in diamond, as well as for magnetometry and MW imaging applications.

## Methods

### Measurements

A pulsed laser diode at a wavelength of *λ* = 520 nm was driven by a high speed driver at the peak power of 70 mW. The photoluminescence arising from the diamond substrate was collected by an objective lens 100 ×  with an NA 0.73 and a working distance of 4.7 mm, and was focused onto a cooled scientific CMOS camera. The static magnetic field was applied by two Nd_2_Fe_14_B permanent magnets aligned along the [111] direction. We used a MW planar ring antenna with a single-loop coil surrounding a circular hole with a radius of 0.5 mm; the resonance frequency in the range of 2.7–3.1 GHz and an input impedance matched to 50 *Ω*^23^. Microwave pulses were created by a signal generator (SMC100A, Rhodes Schwarz), a microwave switch (ZFSWA2-63DR+, Mini-Circuits), and an arbitrary wave generator (33622A, Keysight), and were amplified by a power amplifier (ZHL-16W-43+, Mini-Circuits).

### Samples

We used a (100) CVD type IIa ultra-pure diamond substrate with a size of 2.0 × 2.0 × 0.5 mm^3^. After implantation of ^15^N${}_{2}^{+}$ ions at 10 keV with a dose of 2 × 10^12^ – 2 × 10^13^ cm^−2^^28^, the diamond substrate was annealed at 800 ^∘^C and treated by acid. The diamond substrate was cut along the [110] direction, aligned along the *x*-direction in the laboratory frame. The gold structures were prepared by electron beam evaporation of 10 nm of Ti and 110 nm of Au on a Si substrate with a size of 10 × 10 × 0.5 mm^3^.

### FDTD simulations

The FDTD simulations were performed using the open source program *OpenFDTD*^29^. The Maxwell’s equations were solved numerically in the time domain by the leapfrog integration method. The time step was 0.68 fs. The size of the calculated space was 150 × 150 × 100 mm^3^. The number of the cells was 369 × 320 × 260. Variable mesh size was used, and the minimum size of the mesh was 0.5 *μ*m. An absorbing boundary condition of a perfectly matched layer was used.^29^ The material parameters used in the calculations were the relative permittivity of Si and diamond of 11.20 and 5.68, respectively, and the conductivity of Au of 4.70 × 10^7^ S/m. The source of the MW field was a double dipole antenna located at a distance of 30 mm from the resonators. The dipoles were phase-shifted by 180^∘^.

## Data Availability

The data supporting the findings of this study are available from the corresponding author on reasonable request.
